# Pigmented Actinic Keratosis: Case Report and Review of an Uncommon Actinic Keratosis Variant that can Mimic Melanoma

**DOI:** 10.7759/cureus.4721

**Published:** 2019-05-22

**Authors:** Boya Abudu, Antoanella Calame, Philip R Cohen

**Affiliations:** 1 Internal Medicine, Kaiser Permanente Oakland Medical Center, Oakland, USA; 2 Dermatology, Compass Dermatopathology, Inc., San Diego, USA; 3 Dermatology, San Diego Family Dermatology, National City, USA

**Keywords:** actinic, immunoperoxidase, keratosis, lentigo, maligna, malignant, melanoma, pigmented, solar, spreading

## Abstract

Pigmented actinic keratosis is an uncommon variant of actinic keratosis that can mimic melanocytic lesions. A 54-year-old man who presented with a dark lesion on his nasal tip is described; biopsy of the lesion revealed a pigmented actinic keratosis that was treated with cryotherapy using liquid nitrogen. Pigmented actinic keratoses typically appear on sun-exposed areas of the skin as flat hyperpigmented lesions that grow in a centrifugal pattern. Dermoscopy reveals one or more pseudonetworks with hyperpigmented dots or globules. Histopathology shows atypical keratinocytes in the epidermal basal layer and increased melanin content in the epidermis and dermis. Treatment options include liquid nitrogen cryotherapy for solitary lesions and curettage, 5-fluorouracil, imiquimod, ingenol mebutate, photodynamic therapy, or superficial peels for extensive lesions.

## Introduction

Pigmented actinic keratosis is an uncommon clinical variant of actinic keratosis [1-18]. This precancerous lesion can mimic not only melanocytic lesions but also other epithelial tumors [7-8,16-18]. The clinical and pathologic features of an actinic keratosis on the nasal tip of a man are described and the characteristics of this unique lesion are reviewed.

## Case presentation

A 54-year-old man presented for evaluation of a new dark lesion on his nose. The asymptomatic lesion had been present for more than a year and had progressively increased in size.

**Figure 1:**
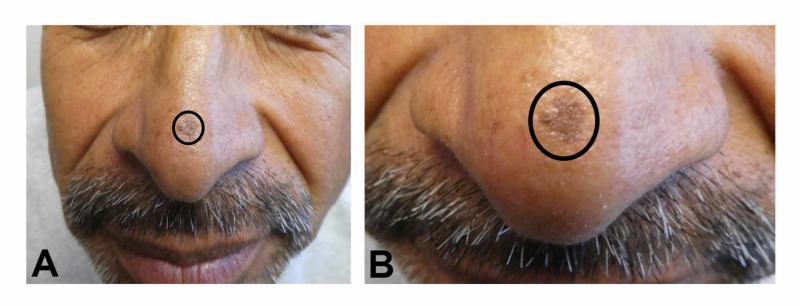
Clinical presentation of a pigmented actinic keratosis on the nasal tip of a 54-year-old man Distant (A) and closer (B) views of a man’s nose show a pigmented actinic keratosis presenting as a 5 x 5-mm black patch

Clinical examination of his face showed a 5 x 5-mm black patch on the nasal tip (Figure 1). Complete evaluation of his entire body did not show any similar lesions. A 2-mm punch biopsy was performed.

**Figure 2:**
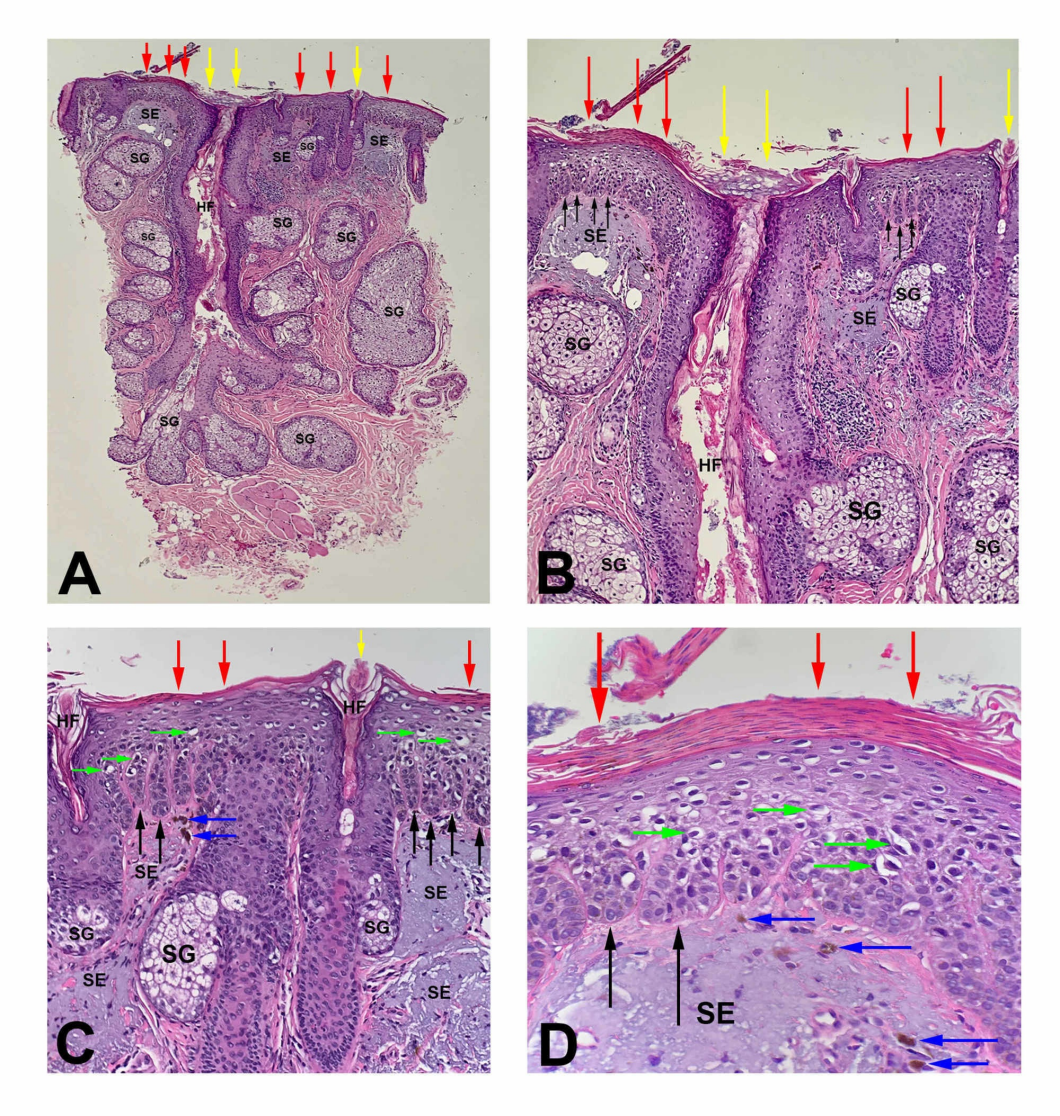
H&E stained sections of a pigmented actinic keratosis Low (A) and higher (B-D) views of a pigmented actinic keratosis. There is alternating parakeratosis (red arrows) and orthokeratosis (yellow arrows) overlying the epithelium. There is acanthosis and elongation of hyperpigmented rete ridges (black arrows) which contain cells with enlarged and atypical nuclei. There are individual melanocytes (green arrows) along the basal layer of the epidermis. There are prominent HFs with their associated SG; the cells in the basal layer of the follicular epithelium are not atypical. In the upper dermis, there is extensive SE and several melanophages (blue arrows; H&E: A, x2; B, x4; C, x10; D, x20). H&E, hematoxylin and eosin; HF, hair follicles; SG, sebaceous glands; SE, solar elastosis

Microscopic examination of hematoxylin and eosin stained sections showed a proliferation of atypical keratinocytes in the lower layers of the epidermis with overlying parakeratosis (Figure 2). There was sparing of adnexal epithelium. However, the lesion extended to the lateral margins of the specimen.

**Figure 3:**
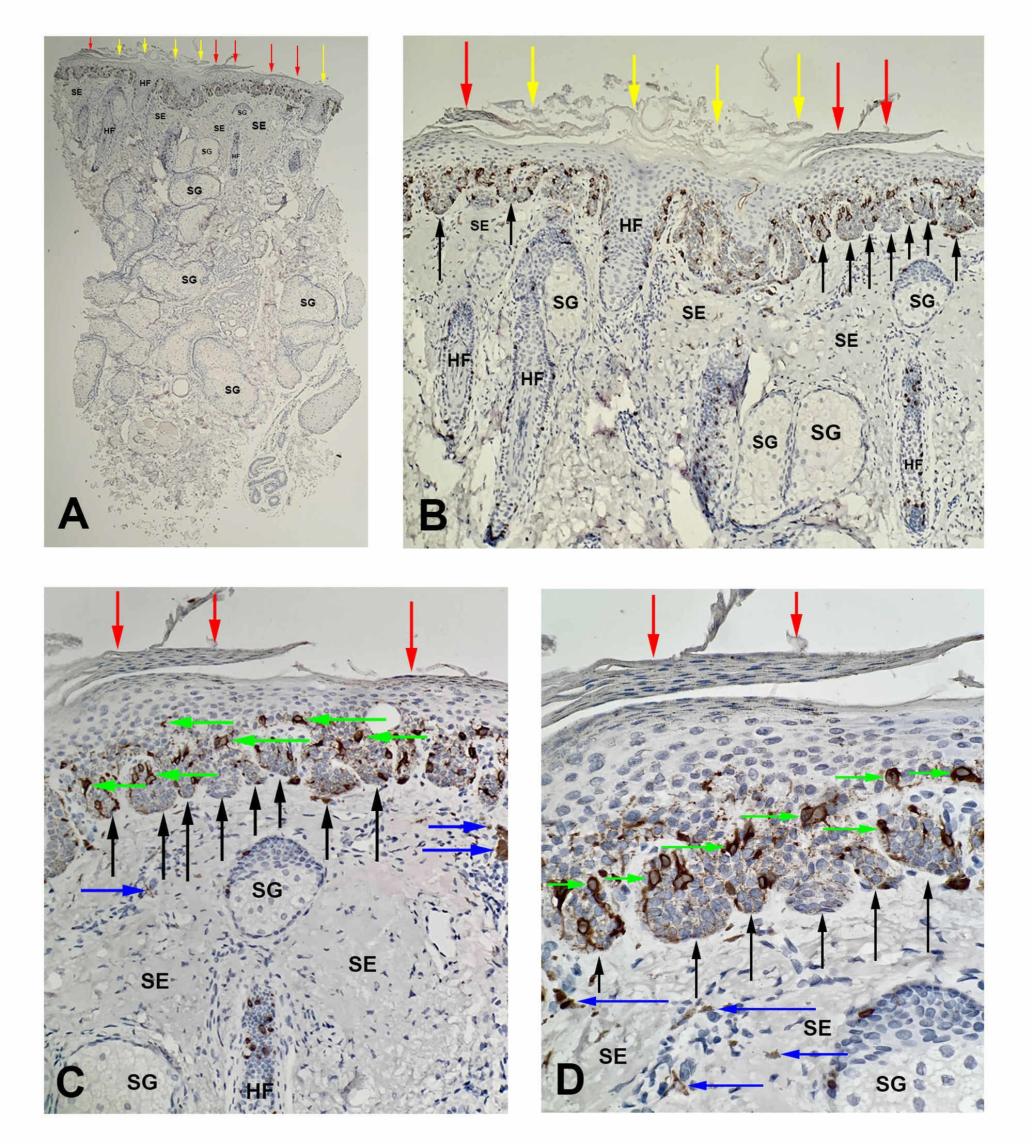
MART-1 stained sections of a pigmented actinic keratosis Low (A) and higher (B-D) views of a pigmented actinic keratosis. MART-1 stains individual melanocytes (green arrows) along the basal layer of the epidermis. The atypical cells in the basal layers of the elongated rete ridges (black arrows) do not show positive staining. In addition to these features, similar features noted on the hematoxylin and eosin sections can also be observed: alternating parakeratosis (red arrows) and orthokeratosis (yellow arrows), acanthosis, prominent HFs with their associated SG, and SE with occasional melanophages (blue arrows) in the dermis (MART-1 immunoperoxidase: A, x2; B, x4; C, x10; D, x20). MART-1, melanoma antigen recognized by T cells 1; HF, hair follicles; SG, sebaceous glands; SE, solar elastosis

**Figure 4:**
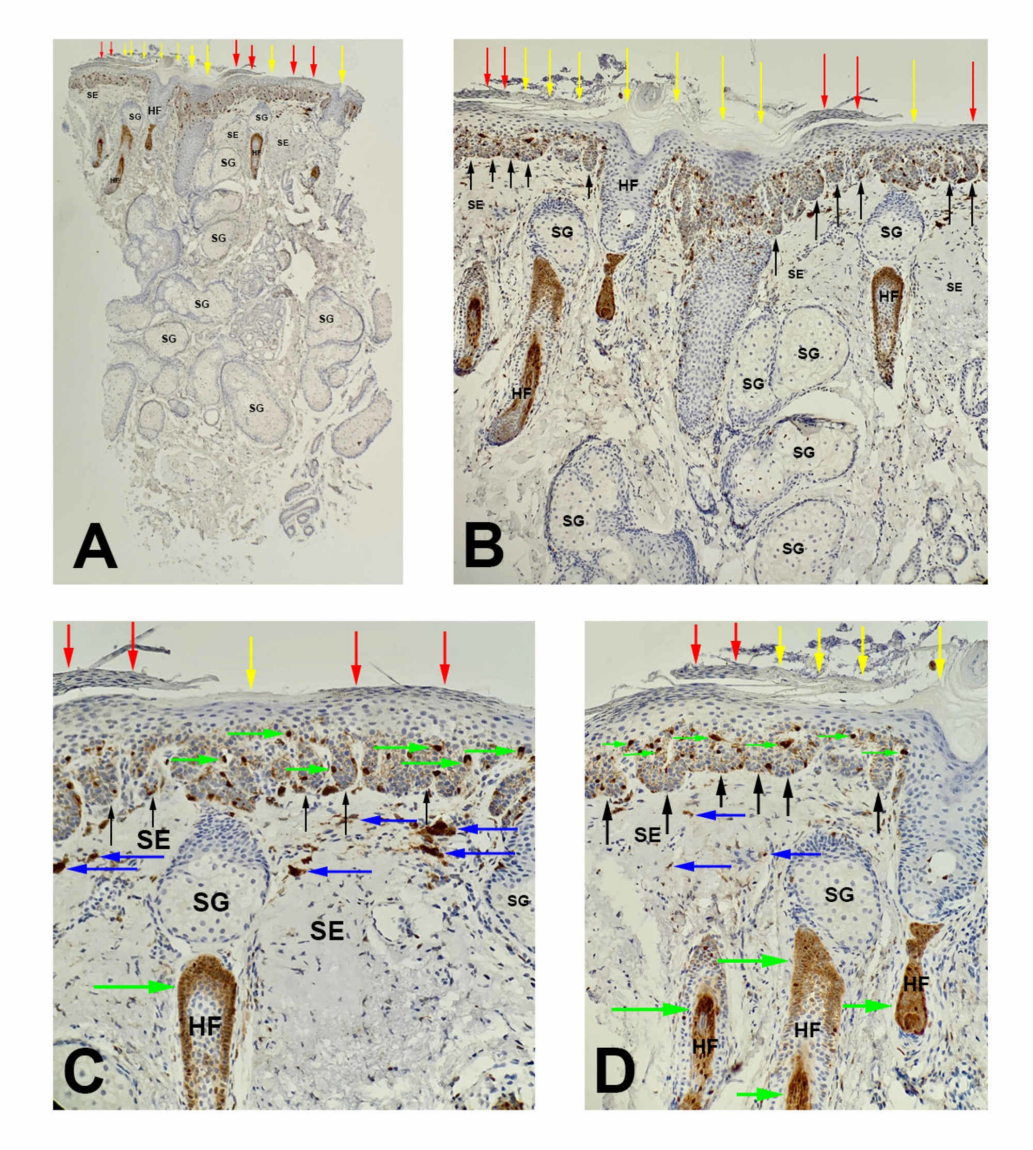
MiTF stained sections of a pigmented actinic keratosis Low (A) and higher (B-D) views of a pigmented actinic keratosis. MiTF shows positive staining of melanocytes (green arrows) in the lower layers of the epidermis and within the hair follicles (HF); the atypical cells that comprise the elongated rete ridges (black arrows) do not show positive staining for MiTF. In addition to these features, similar features noted on the hematoxylin and eosin sections can also be observed: alternating parakeratosis (red arrows) and orthokeratosis (yellow arrows), acanthosis, prominent HF with their associated SG, and SE with occasional melanophages (blue arrows) in the dermis (MiTF immunoperoxidase: A, x2; B, x4; C, x10; D, x20). MiTF, microphthalmia-associated transcription factor; HF, hair follicles; SG, sebaceous glands; SE, solar elastosis

Immunohistochemical stains were performed. MART-1 and MiTF did not stain the atypical cells. Therefore, an atypical melanocytic proliferation was excluded (Figures 3, 4).

Correlation of the history, clinical presentation, and pathologic findings established the diagnosis of a pigmented actinic keratosis. The residual lesion was treated with cryotherapy using liquid nitrogen. The lesion completely resolved after treatment; follow-up examinations demonstrate that it has not recurred.

## Discussion

Actinic keratosis is an epithelial lesion characterized by atypical keratinocytes predominately restricted to the lower layers of the epidermis; some of these lesions have the potential to develop into cutaneous malignancies such as squamous cell carcinoma. Although most actinic keratoses present as erythematous scaly plaques, an uncommon clinical variant manifests as a pigmented lesion. In this setting, the diagnosis may not be suspected.

Pigmented actinic keratosis was initially described by James et al. in 1978 [1]. They reported 10 lesions on the face of nine Caucasian patients and one patient of Egyptian origin. The lesions appeared as smooth, verrucous, or scaly plaques. Two of the lesions had progressed into invasive pigmented squamous cell carcinomas. The patients were treated with cryotherapy using liquid nitrogen, excision, or topical 5-fluorouracil.

Subsequently, this variant of actinic keratosis was described by Subrt et al. in 1983 [2]. They observed four patients who demonstrated spreading lesions that similarly varied in texture and size. While the diagnosis of pigmented actinic keratosis has increased since these reports, it continues to be an uncommon and frequently misdiagnosed premalignant condition.

Pigmented actinic keratoses most commonly present on sun-exposed areas of the skin. In contrast to patients with lighter skin types who commonly present with nonpigmented actinic keratoses, patients with darker skin types are more likely to develop pigmented lesions [3]. Pigmented actinic keratoses present as papules or plaques that are rough or scaly and vary in color from brown to gray, typically with a dull surface. Lesions range in size from less than 1 cm to greater than 5 cm in diameter [4]. Most pigmented actinic keratoses demonstrate progressive lateral growth, which has been described as either “spreading” or “centrifugal” [2]. Smaller pigmented actinic keratoses can appear as multiple similar-appearing lesions. Larger pigmented actinic keratoses are usually solitary. Irritation or pruritus are common presenting symptoms while bleeding, ulceration, and pain are less frequently reported [4].

A characteristic dermoscopic feature of a nonpigmented actinic keratosis is a “strawberry-pattern”; this consists of a superficial pseudonetwork containing a pink to red vascular plexus between adnexal openings, each surrounded by a white targetoid halo. Yellow keratotic plugs are frequently found within the hair follicles. Visualization of underlying features may be difficult when hyperkeratosis is present; in this setting, dermoscopy may demonstrate a pink or yellow hue without a distinct pseudonetwork. The superficial scale may also present on dermoscopy [5-6].

The dermoscopic features of a pigmented actinic keratosis also contain a pseudonetwork but more typically presents as a brown region of hyperpigmentation. Such pseudonetworks may appear as a solitary unit or present as more than one intermingling or discontinuous pseudonetworks. Tan, gray, or black dots and globules and the rhomboidal structures may occasionally present within this network [7].

Biopsy may be necessary to differentiate pigmented actinic keratosis from lentigo maligna since they share many dermoscopic features. Dermoscopic examination of lentigo maligna will demonstrate asymmetric pigmented follicular openings, rhomboidal structures, and black blotches and streaks. However, features such as pseudonetworks and hyperpigmented globules are also present in lentigo maligna; therefore histopathology is the gold standard for establishing a diagnosis [8].

The pathologic features of pigmented actinic keratosis have been defined with routine hematoxylin and eosin staining, immunoperoxidase staining, and electromicroscopy. The histopathology of nonpigmented actinic keratosis shows solar elastosis in the dermis with cytologic atypia of the basal keratinocytes and parakeratosis in which there is sparing of the follicular epithelium. Foci of acantholysis and mitotic figures are additional features of nonpigmented actinic keratosis. In addition to the features observed in nonpigmented actinic keratoses, pigmented actinic keratosis will also show increased melanin within the epidermis and dermis [7].

Chung et al. reviewed 167 cases of pigmented actinic keratosis to characterize their histopathologic features [7]. The investigators demonstrated that some of the lesions with a clinical diagnosis of pigmented actinic keratosis revealed a nonpigmented actinic keratosis adjacent to or coexisting with a pigmented lesion in 81% of the cases. However, they also observed that nearly one-fifth of the lesions (19%) were bona fide pigmented actinic keratoses.

When the investigators characterized the 136 collision tumors containing a nonpigmented actinic keratosis, the most common concurrent lesion was a solar lentigo (121 cases). However, seborrheic keratosis (18 cases), melanoma in situ (three cases), or lichen planus-like keratosis (two cases), were also noted; seven collision tumors had more than one pigmented lesion―most commonly a seborrheic keratosis and a solar lentigo. The high frequency of collision tumors containing nonpigmented actinic keratosis suggests that there would be a large spectrum of clinical and dermatoscopic presentations.

Immunoperoxidase staining can be helpful in differentiating actinic keratosis lesions and melanocytic lesions. Actinic keratosis and squamous cell carcinoma demonstrate positive staining with cytokeratin. In contrast, melanocytic lesions can be identified using stains against melanocyte antigens (Table 1) [9-13].

**Table 1:** Immunoperoxidase stains to differentiate pigmented actinic keratosis from melanocytic neoplasms HMB-45, Human Melanoma Black 45; MART-1, melanoma antigen recognized by T cells 1; MiTF, microphthalmia-associated transcription factor; Ref, References; SOX10, Sry-related HMG-BOX gene 10

Peroxidase Stain	Features	Ref
HMB-45	Stains the 10-kDa cytoplasmic glycoprotein of the glycoprotein 100 premelanosome complex. This marker can sometimes help distinguish invasive melanoma from melanocytic nevi.	[9]
MART-1/Melan-A	Melanosome-specific protein that results in cytoplasmic staining, resulting in staining of all cells derived from melanocytes. Does not distinguish benign melanocytic nevi from premalignant melanocytic lesions or melanoma.	[10]
MiTF	Marker of all cutaneous melanocytes that stains the transcription factor responsible for regulation of the pigmentation enzyme genes tyrosinase and tyrosinase-related proteins 1 and 2. There is also expression of MiTF in hair follicles of normal skin samples; therefore, MiTF stains melanocytes in the epidermis as well as hair follicles. In the epidermis, this nuclear immunostain is highly sensitive and specific to melanocytes.	[12]
S100	Stains the calcium-binding protein found on the 10-kDa cytoplasmic glycoprotein region of the transmembrane complex of various cell types, including melanocytes and neural crest-derived cells (such as Schwann cells, glial cells). The name S100 derives from the observation that the protein is soluble in 100% saturated ammonium sulfate solution.	[9, 11]
SOX10	Stains the transcription factor responsible for the specification of neural crest cells and maintenance of melanocytes and Schwann cells.	[13]

Initially, S100 was used. More recently, newer markers have been incorporated such as melanoma antigen recognized by T cells 1 (MART-1), microphthalmia-associated transcription factor (MiTF), and Sry-related HMG-BOX gene 10 (SOX-10) [14]. Similar to our patient, the atypical cells are negative for these melanocytic markers in lesions of pigmented actinic keratosis.

Electron microscopy of pigmented actinic keratosis demonstrates an increased level of melanosomes in the keratinocytes in the basal layer of the epidermis; these melanosomes are most prominently distributed within the cytoplasm of dendritic processes. Melanosomes appear as either single units or complexes; the melanosome complexes are usually membrane-bound and in varying stages of degradation. Langerhans’ cells and macrophages may also present with large numbers of melanosome complexes [15].

The differential diagnosis of pigmented actinic keratosis includes lentigo maligna, lichen planus-like keratosis, and solar lentigo (Table 2) [7-8,16-18]. Since their clinical presentation can be identical, dermoscopy and microscopic features are helpful to differentiate the lesions. However, since the findings on dermoscopy may overlap and since a pigmented actinic keratosis may indeed represent a collision tumor, a biopsy may be required to establish the diagnosis [8,19]. A partial biopsy should be avoided since a collision tumor containing an adjacent melanoma in situ may be missed.

**Table 2:** Clinical differential diagnosis of pigmented actinic keratosis DEJ, dermoepidermal junction; LM, lentigo maligna; LPLK, lichen planus-like keratosis; PAK, pigmented actinic keratosis; SL, solar lentigo

Condition	Clinical Presentation	Dermoscopy	Pathology	Ref
LM	Variegated brown or gray macule or patch with irregular borders	Pseudonetwork involving asymmetric follicular openings with hyperpigmented rim, gray or black dots and globules; rhomboidal structures	Atypical melanocytic hyperplasia at DEJ with variable adnexal involvement; nesting of atypical melanocytes; occasional pagetoid spread of melanocytes	[8, 18]
LPLK	Erythematous, violaceous, or hyperpigmented papule or plaque with or without adherent scale	Diffuse brown and gray granules that may coalesce to form globules, streaks, or rhomboid-like structures	Hyperkeratosis with focal epidermal parakeratosis, acanthosis, variable hypergranulosis, basal layer vacuolization, and lichenoid interface dermatitis with lymphocytes and histiocytes. Colloid bodies are present. Eosinophils and plasma cells are occasionally present	[8, 16]
PAK	Brown or gray rough papule with ill-defined borders with or without adherent scale	Discontinuous brown pseudonetwork and keratin plugs	Solar elastosis, cytologic atypia of basal keratinocytes, and parakeratotic sparing of follicular epithelium. Foci of acantholysis and mitotic figures. Increased melanin within the epidermis and dermis	[7, 8]
SL	Brown or black macule or patch with smooth or irregular borders	Pseudonetwork; yellow opaque areas, horny pseudocysts, milia-like cysts, cerebriform structures, moth-eaten border, sharp border (jelly sign)	Epidermal hyperplasia and hyperpigmentation of the basal layer of the epidermis	[8, 17]

The etiology of pigmented actinic keratosis is unclear; however, several theories have been proposed for its presentation. The number of melanocytes in the basal layer of the epidermis in pigmented actinic keratosis is the same as nonpigmented actinic keratosis; however, electron microscopy studies demonstrate enhanced melanosome formation in epithelial keratinocytes [15]. It has also been proposed that, in some cases, pigmented actinic keratosis is a collision tumor. Indeed, concurrent lesions involving a nonpigmented actinic keratosis and either lichen planus-like keratosis, melanocytic nevus, melanoma in-situ, seborrheic keratosis, and a solar lentigo have been reported [7,19]. 

Treatment options for pigmented actinic keratosis are the same as that for nonpigmented actinic keratosis. Lesions may be treated using cryotherapy with liquid nitrogen; this is usually an effective approach, particularly if the pigmented actinic keratosis is a solitary lesion. Topical therapy ― with either 5-fluorouracil, imiquimod, or ingenol ― curettage, photodynamic therapy, and superficial peels may be used when there are multiple lesions [20].

## Conclusions

Pigmented actinic keratosis is a variant of actinic keratosis that occurs less commonly. In contrast to presenting as an erythematous plaque, its appearance can mimic a pigmented lesion (lentigo maligna or solar lentigo) or a keratinocytic lesion (lichen planus-like keratosis). Dermoscopy may be helpful in suggesting the correct diagnosis. However, dermoscopic features of a pigmented keratosis can overlap with those of the pigmented lesions in the differential diagnosis. Also, some pigmented actinic keratoses actually represent collision tumors consisting of a nonpigmented actinic keratosis and a primary melanocytic lesion. Therefore, microscopic examination of a lesional biopsy may be necessary to establish the diagnosis. Although the pathogenesis has not been established, it has been suggested that increased melanosome formation as well as the presence of nonpigmented actinic keratosis in collision tumors lead to its development, particularly in patients with darker skin types. The management of a pigmented actinic keratosis is the same as nonpigmented actinic keratosis. Liquid nitrogen cryotherapy should be considered for the treatment of a solitary lesion. Field treatment with 5-fluorouracil, curettage, imiquimod, ingenol mebutate, photodynamic therapy, or superficial peels may be appropriate when there are multiple lesions within the same area.
